# DNA in serum extracellular vesicles is stable under different storage conditions

**DOI:** 10.1186/s12885-016-2783-2

**Published:** 2016-09-23

**Authors:** Yang Jin, Keyan Chen, Zongying Wang, Yan Wang, Jianzhi Liu, Li Lin, Yong Shao, Lihua Gao, Huihui Yin, Cong Cui, Zhaoli Tan, Liejun Liu, Chuanhua Zhao, Gairong Zhang, Ru Jia, Lijuan Du, Yuling Chen, Rongrui Liu, Jianming Xu, Xianwen Hu, Youliang Wang

**Affiliations:** 1 Laboratory of Cell Engineering, Institute of Biotechnology, Beijing, China; 2 Affiliated Hospital Cancer Center, Academy of Military Medical Sciences, Beijing, China; 3 Department of Ultrasonics, People’s Hospital, Donggang District, Rizhao, Shandong Province China

**Keywords:** Extracellular vesicles, DNA, Stability, Different conditions

## Abstract

**Background:**

Extracellular vesicles (EVs), including exosomes, microvesicles, and apoptotic bodies, can be secreted by most cell types and released in perhaps all biological fluids. EVs contain multiple proteins, specific lipids and several kinds of nucleic acids such as RNAs and DNAs. Studies have found that EVs contain double-stranded DNA and that genetic information has a certain degree of consistency with tumor DNA. Therefore, if genes that exist in exosomes are stable, we may be able to use EVs genetic testing as a new means to monitor gene mutation.

**Methods:**

In this study, EVs were extracted from serum under various storage conditions (4 °C, room temperature and repeated freeze-thaw). We used western blotting to examine the stability of serum EVs. Then, we extracted DNA from EVs and tested the concentration changing under different conditions. We further assessed the stability of EVs DNA s using polymerase chain reaction (PCR) and Sanger sequencing.

**Results:**

EVs is stable under the conditions of 4 °C (for 24 h, 72 h, 168 h), room temperature (for 6 h, 12 h, 24 h, 48 h) and repeated freeze-thaw (after one time, three times, five times). Also, serum DNA is mainly present in EVs, especially in exosomes, and that the content and function of DNA in EVs is stable whether in a changing environment or not. We showed that EVs DNA stayed stable for 1 week at 4 °C, 1 day at room temperature and after repeated freeze-thaw cycles (less than three times). However, DNA from serum EVs after 2 days at room temperature or after five repeated freeze-thaw cycles could be used for PCR and sequencing.

**Conclusions:**

Serum EVs and EVs DNA can remain stable under different environments, which is the premise that EVs could serve as a novel means for genetic tumor detection and potential biomarkers for cancer diagnostics and prognostics.

## Background

Extracellular vesicles (EVs) are cell-derived membrane vesicles, including exosomes, microvesicles, and other types of membrane vesicles [1]. The best studied of these vesicles are exosomes. Exosomes are nanometer-sized EVs of endocytic origin that are secreted by most cell types, under physiological and pathological conditions upon fusion of multivesicular bodies (MVBs) with the plasma membrane (PM) [2–5]. Recent studies have shown that exosomes can be released in many and perhaps all biological fluids, including blood [6], urine [7], cerebrospinal fluid [8] breast milk [9], malignant ascites [10, 11], saliva [12], tears [13], nasal secretions [14], semen [15], amniotic fluid [16], bronchoalveolar lavage fluid [17] and culture medium supernatant in cell cultures [3]. Although the term exosome was originally raised in 1981 [18], interest in these EVs has increased dramatically in the last few years, after researchers found that they predominantly contain RNA, proteins, and lipids [19], which can reflect the functionality of the host cell and possess molecular signatures or footprints resembling the cell from which they were secreted [20]. Therefore, the EVs has emerged as an important novel mediator in facilitating intercellular communication by regulatory molecules in its cargo and inducing physiologic and genetic changes in targeted cells [21].

Compared with different types of RNA, such as messenger RNA (mRNA), microRNA, and noncoding RNA (ncRNA), which have been shown in the Exocarta database [19], less is known about EVs DNA content, although some types of DNA have been reported, such as single-stranded DNA (ssDNA) [22], mitochondrial DNA (mtDNA) [23] and plasmid DNA (pDNA) [24]. The question remains as to whether the DNA cargo is randomly sorted or if it is systematically packed into EVs. A recent report by Mark et al. found that exosomes contain double-stranded DNA (dsDNA)and that exosome DNA can serve as a novel biomarker for cancer detection [25]. Furthermore, an increasing number of studies have emerged showing that different types of DNA in EVs may be associated with various biological functions [26–28].

It is well-known that exosomes contain dsDNA. Also, exosomal DNA (exoDNA) comprises the entire genome, which spans all chromosomes [29] and carries on the mutational status of malignant parental cells [25]. The question is whether exosomes can be utilized as scapegoats for tumor cells to harbor tumor-specific genetic mutations similar to other sources of circulating tumor DNA (ctDNA) [30]. If these assumptions are established, the feasibility of exosomes as new tumor detection indicators will then became a critical question. As we have described above, EVs are widely distributed in various body fluids systems, which make them more accessible than tumor cells. Moreover, if EVs prove to have considerable stability in vitro, they will become the most promising and available samples for cancer genetic detection. In the present study, we extracted DNA of EVs from the blood of cancer patients under different conditions, and its stability was evaluated.

## Methods

### Serum samples

A collection of serum samples was approved by the local ethics committee of the Affiliated Hospital, Academy of Military Medical Sciences. A written informed consent for the serum sampling was obtained preoperatively from all patients with the disclosure of planned analyzes regarding potential prognostic markers.

### Isolation of EVs from serum

Peripheral blood samples from patients with metastatic colorectal cancer (mCRC) were centrifuged at 3 000 g for 15 min at ambient temperature (within 2 hours of the blood drawing) to remove cells and cell debris. EVs were isolated from the serum using ExoQuick reagent (System Biosciences, Palo Alto, California, USA) [31] or PureExo® Exosome Isolation Kit (101 Bio, Palo Alto, California, USA). For ExoQuick isolation, 120 μl ExoQuick reagents was added to 500 μl serum and precipitated 30 min at 4 °C. Precipitated samples were centrifuged at 1 500 g for 30 min at room temperature. 120 μl ExoQuick reagent was added to the supernatant and precipitated at 4 °C for another 30 min. Precipitated samples were centrifuged at 1 500 g for 30 min at room temperature. The pooled EVs pellets were dissolved in the appropriate buffer for DNA or protein analysis. For PureExo® Exosome Isolation Kit, EVs were extracted from 500 μl serum according to the instructions in step.

### Transmission electron microscopy

EVs samples (10 μl) resuspended in phosphate buffer saline (PBS) were applied to 300 mesh carbon-coated copper grids for 10 min. Excess samples were blotted with filter paper and then negatively stained with 10 μl of a 2 % aqueous uranyl acetate solution for 3 min. Stain was blotted dry from the grids with filter paper, and samples were allowed to dry. Samples were then examined in a HITACHI H-7650 transmission electron microscope (HITACHI, Tokyo, Japan) at an accelerating voltage of 80 kV.

### Proteins extraction and quantification

EVs samples from 500 μl of serum were lysed in 200 μl RIPA buffer [65 mM Tris-HCl pH 7.4, 150 mM NaCl, 1 mM EDTA, 1 % NP-40, 0.25 % sodium deoxycholate, protease inhibitors cocktail (Roche Diagnostics, Mannheim, Germany), and phosphatase inhibitor (Biomed, Beijing, China)] and incubated at 0 °C for at least 30 min. Incubated specimens were then centrifuged at 12,000 rpm for 30 min at 4 °C. Protein concentration was analyzed by the Pierce™ BCA protein assay kit (Thermo Fisher Scientific, Wilmington, Delaware, USA).

### Western blotting

A total of 10 μg exosomal proteins were loaded on a regular SDS-PAGE gel. Following electrophoresis, gels were transferred to PVDF (polyvinylidene fluoride) membranes (Millipore, Billerica, USA) for western blotting. Membranes were blocked with 5 % nonfat dry milk dissolved in 1 × PBST (PBS plus 0.2 % v/v Tween-20) for 2 h at room temperature, probed with antigen-specific antibodies overnight at 4 °C and washed with 1 × PBST solution three times. Afterward, membranes were incubated with secondary antibodies for 1 h at room temperature and washed with 1 × PBST solution three times. Blots were developed with chemiluminescent reagents from Pierce (Thermo Fisher Scientific).

### DNA isolation

Before the DNA isolation, samples were treated with 2000 U/ml DNase I (New England BioLabs, Frankfurt, Germany) for 2 h at 37 °C to remove possible nucleic acid contaminants. After treatment, the enzymes were heated to the condition of inactivation at 75 °C for 10 min (this step reaction was only used to verify the distribution of serum DNA). First, total DNA was extracted from EVs by using DNA lysis buffer [0.5 % SDS, 0.05 M EDTA, 0.01 M Tris-HCl pH 8.0, 0.1 M NaCl, 200 μg/ml Protease K (Amresco, Solon, Ohio, USA)]. A total of 400 μl DNA lysis buffer was added to each tube of EVs. After mixing, the samples were incubated for 24 h at 55 °C. The serum that EVs had been extracted out of did not need the process above. Second, deproteinization was performed using a balance of phenol and chloroform. Third, DNA was precipitated using 3 M CH_3_COONa, glycogen and absolute ethanol for 24 h at −20 °C and resuspended DNA with TE (0.001 M EDTA, 0.01 M Tris-HCl pH 8.0) at 37 °C for at least 16 h. The EVs DNA was quantified on a Nano Drop ND-2000 Spectrophotometer (Thermo Fisher Scientific).

### PCR analysis and sequencing

The polymerase chain reaction (PCR) analysis was performed with preamplified DNA products from EVs and serum disposed of EVs samples from patients with different conditions by using specifically designed primers (Table 1). Each experiment has repeated a minimum of three times. The PCR reaction mixture contained 25 μl 2 × Taq Master Mix (Sino Bio Technologies, Shanghai, China), 2 μl 1 × primer, 4 μl resuspended DNA and double-distilled water up to 50 μl (100 mM dNTP mix, 10 mM of each primer, 5 U/ml of Taq 5000 DNA polymerase). PCR was performed in a C1000™ Thermal Cycler (Bio-Rad, Hercules, California, USA) and the thermal cycles were as follows: 1 cycle of 2 min at 94 °C, 30 cycles for 30 s at 94 °C, 30 s at 58 °C and 30 s at 72 °C, and 5 min at 72 °C. The resulting PCR products were validated by electrophoresis using 3 % agarose (Amresco) gels. PCR products were sent to Invitrogen Biotechnology to conduct the sequencing analysis.

**Table 1:** Primers used in PCR reactions

Gene	Forward primer	Reverse primer
*KRAS*	5'-CCTGCTGAAAATGACTGAATATA-3'	5'-TCTATTGTTGGATCATATTCGTC-3'
*EGFR*	5'-CTCTCTCTGTCATAGGGACTCTG-3'	5'-AGCAAAGCAGAAACTCACATC-3'
*p53*	5'-CAATGGTTCACTGAAGACCCA-3'	5'-AAGGGACAGAAGATGACAGGG-3'

## Results

### Extraction and identification of serum EVs

**Fig. 1:**
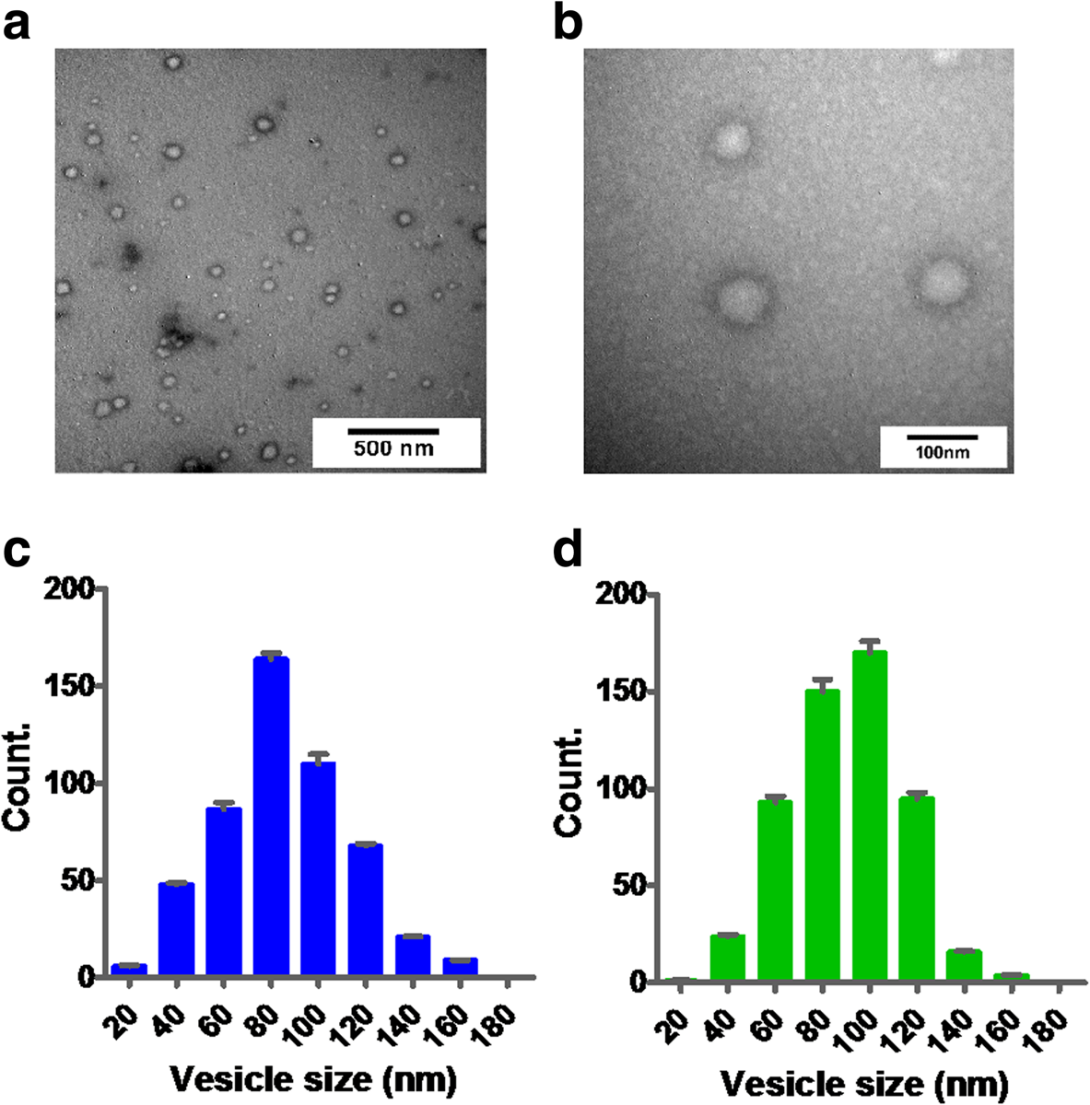
Morphological characterization and size distribution of EVs. **a** Micrograph of serum exosomes with low-magnification TEM; **b** High resolution TEM examination outcomes; **c**, **d** Size distribution of the isolated EVs analyzed by TEM and Image-pro Plus. The EVs were counted three times, and the data represented mean ± SD. EVs were extracted by ExoQuick reagent (**c**) or by PureExo® Exosome Isolation kit (**d**)

**Fig. 2:**
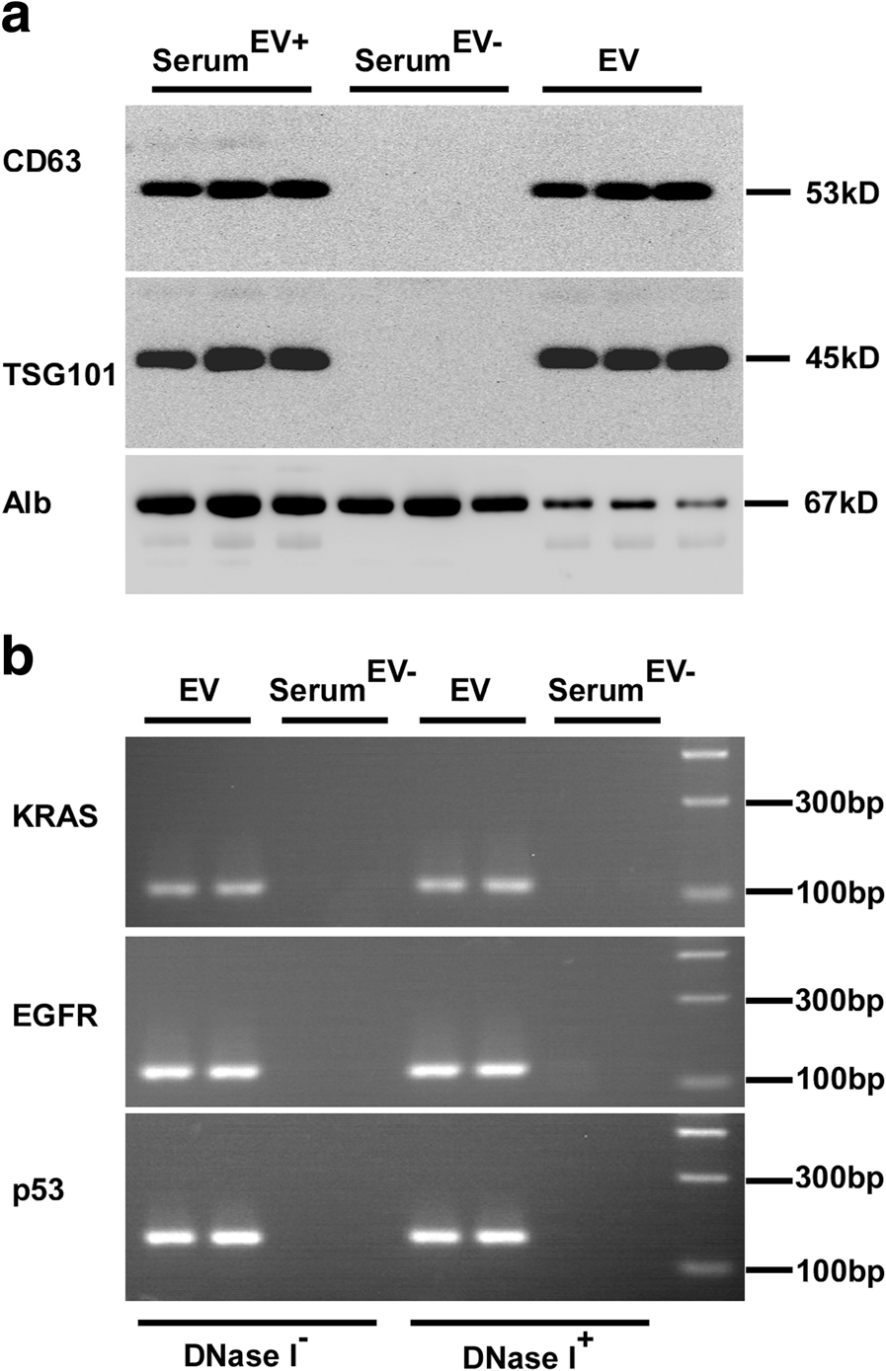
Serum DNA predominates in EVs. **a** EVs were characterized by the exosome specific expression of CD63 and TSG101 by western blotting. **b** EVs DNA isolated from serum pretreated with DNase I or not was detected by PCR amplification. Serum^EV+^ represents EVs-contained serum, Serum^EV-^ represents EVs-depleted serum

We used two different methods to isolate EVs, one of which was the ExoQuick reagent and the other one was Exosome Isolation Kit. Studies have shown that ExoQuick reagent could extract most EVs from blood samples [32, 33] and Exosome Isolation kit could capture most exosomes from serum [34] and that according to the study of 101Bio, using their kit could get 95 % exosome purity. To confirm whether EVs were extracted successfully by the two methods above, harvested serum EVs were analyzed by transmission electron microscopy (TEM) [35]. Particles between 20 and 170 nm were identified (Fig. 1a, b). The average size of EVs observed in 40 captured images by TEM was 90 ± 3 nm; most vesicles were between 40 and 140 nm in diameter, with a peak around 80–100 nm whether the extraction process using ExoQuick reagent (Fig. 1c) or Exosome Isolation Kit (Fig. 1d). There was no significant difference in the size of the EVs obtained by two methods. So we concluded that the main parts of EVs in serum were exosomes, and we used ExoQuick reagent for EVs extraction taking into account the simplicity of the operation in the follow-up test processes. To ensure that the EVs derived from serum were enriched in exosomes, the isolated vesicles were subjected to exosomal marker analysis. The results of the western blotting (Fig. 2a) showed that only serum (serum^EV+^) and EVs contain CD63 and TSG101, which are specific markers of exosomes [36]. However, a serum that had been extracted for EVs (serum^EV-^) did not contain CD63 and TSG101. In addition, albumin, as a ubiquitous protein outside of EVs, mainly existed in serum^EV+^ and serum^EV^. Therefore, we extracted the overwhelming majority of serum EVs (especially exosomes) in the blood sample and proved that the EVs extraction process was successful.

### Serum DNA dominates in EVs

Since serum contains different types of DNA, such as cfDNA, ctDNA and exoDNA [26], the extraction process of EVs DNA must reduce external DNA contamination. Two volunteers were randomly selected, and we took two tubes of identical serum samples from each of them to extract EVs. Before the DNA extraction, one copy of EVs and EVs-depleted serum of each patient were treated extensively with DNase I simultaneously, and the other samples remain undisposed.

We amplified three fragments of *KRAS*, *EGFR* and *p53* (Table 1) from EVs DNA to analyze whether DNA is associated with the outer membrane or inside the EVs. We found that with or without DNase I treatment the pattern from each sample was similar (Fig. 2b). And that the DNA gene amplification results were negative in the serum^EV-^ even the conditions were complete as same as EVs. This result indicated that EV specific DNA predominates in serum DNA.

### EVs are stable in different environments

**Fig. 3:**
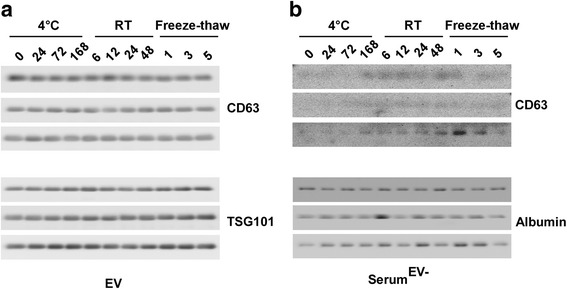
Immunoblot of exosome specific markers in EVs and EVs-depleted serum under different conditions. **a** Immunoblot of CD63 in EVs from the serum of three CRC patients under different conditions. **b** Immunoblot of TSG101 in EVs from the serum of three CRC patients under different conditions. **c** Immunoblot of CD63 in EVs-depleted serum from three CRC patients under different conditions. **d** Immunoblot of albumin in EVs-depleted serum from three CRC patients under different conditions. Freshly isolated serum randomly selected from three advanced CRC patients was stored at 0 h, 4 °C (24 h, 72 h, 168 h), room temperature (6 h, 12 h, 24 h, 48 h) and −80 °C with freeze-thaw (one time, three times, five times)

To assess the impact of disparate storage temperature and different storage time on the stability of EVs, randomly selected freshly isolated serum (after centrifugation of peripheral blood) from three advanced colorectal cancer (CRC) patients was immediately stored at 4 °C (for 24 h, 72 h, 168 h), room temperature (for 6 h, 12 h, 24 h, 48 h) and −80 °C with different freeze-thaw cycles (1 time, 3 times, 5 times). EVs were extracted from the stored serum respectively. The serum volume of each patient under different conditions was consistent. An equal volume of proteins, under various conditions, from either EVs (Fig. 3a, b) or EVs-depleted serum (Fig. 3c) was used for western blotting with antibodies specific for CD63. Our results showed that CD63 and TSG101 remained unchanged in EVs exposed to differing environments. Also, as time went on, we detected trace amounts of CD63 on membranes in EVs-depleted serum. We also incubated the membranes, which had previously been exposed to CD63, with an anti-human albumin antibody. As expected, a clear chromogenic albumin emerged in the EVs-depleted serum films (Fig. 3d). Overall, we concluded that the EVs remained stable in serum exposed to differing environments.

### EVs DNA is stable under different conditions

**Fig. 4:**
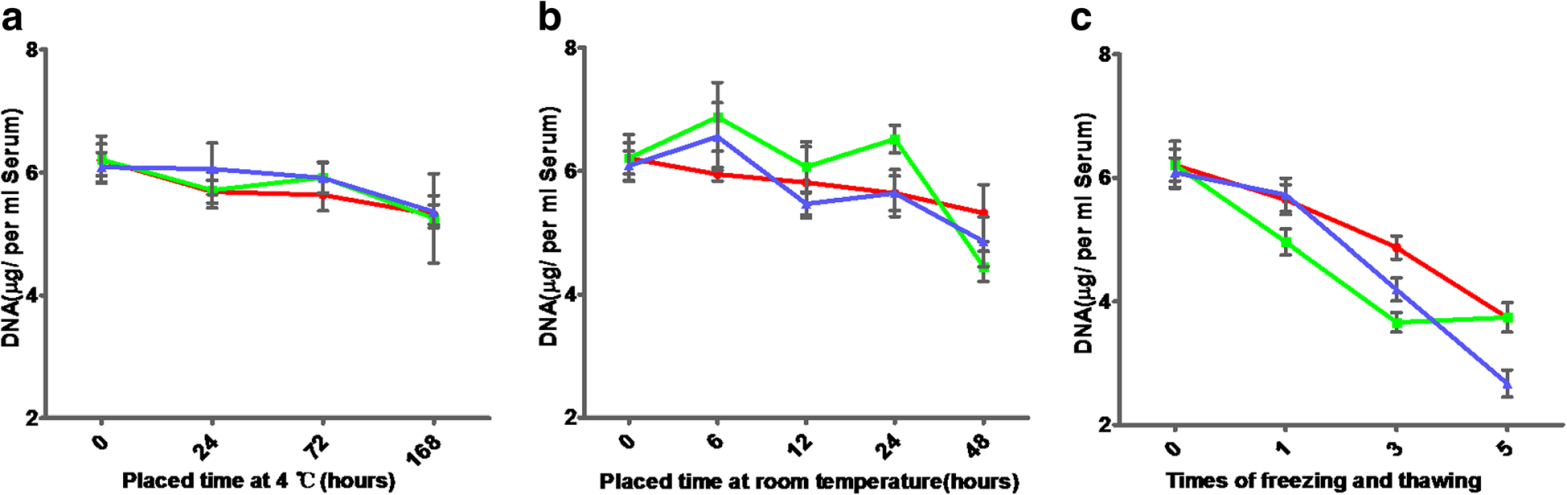
The concentration of EVs DNA extracted from serum under different conditions. **a** The concentration of EVs DNA from the serum of three patients stored at 4 °C for 0, 24, 72 and 168 h. **b** The concentration of EVs DNA from the serum of three patients stored at room temperature for 0, 6, 12, 24 and 48 h. **c** The concentration of EVs DNA from the serum of three patients after repeated freeze-thaw 0, 1, 3 and five times

Since serum DNA dominates in EVs (Fig. 2b), we next asked whether EVs DNA was stable under various conditions. Therefore, we verified the concentration of EVs DNA, which was isolated from the EVs mentioned above. As seen in Fig. 4, we detected variations of EVs DNA concentration among the different patient samples. EVs DNA that was extracted from serum which had been stored at 4 °C was relatively stable (Fig. 4a), in spite of the fact that the concentration of EVs DNA began to decline slowly after 72 h. And apparently, a decrease of concentration from 24 to 48 h was observed in samples that had been stored at room temperature (Fig. 4b). However, EVs DNA that was subjected to freeze-thaw cycles declined dramatically from 0 to 5 times freeze-thaw (Fig. 4c). In comparison to the two cases above, exosomes seemed to be more fragile after freeze-thaw.

**Fig. 5:**
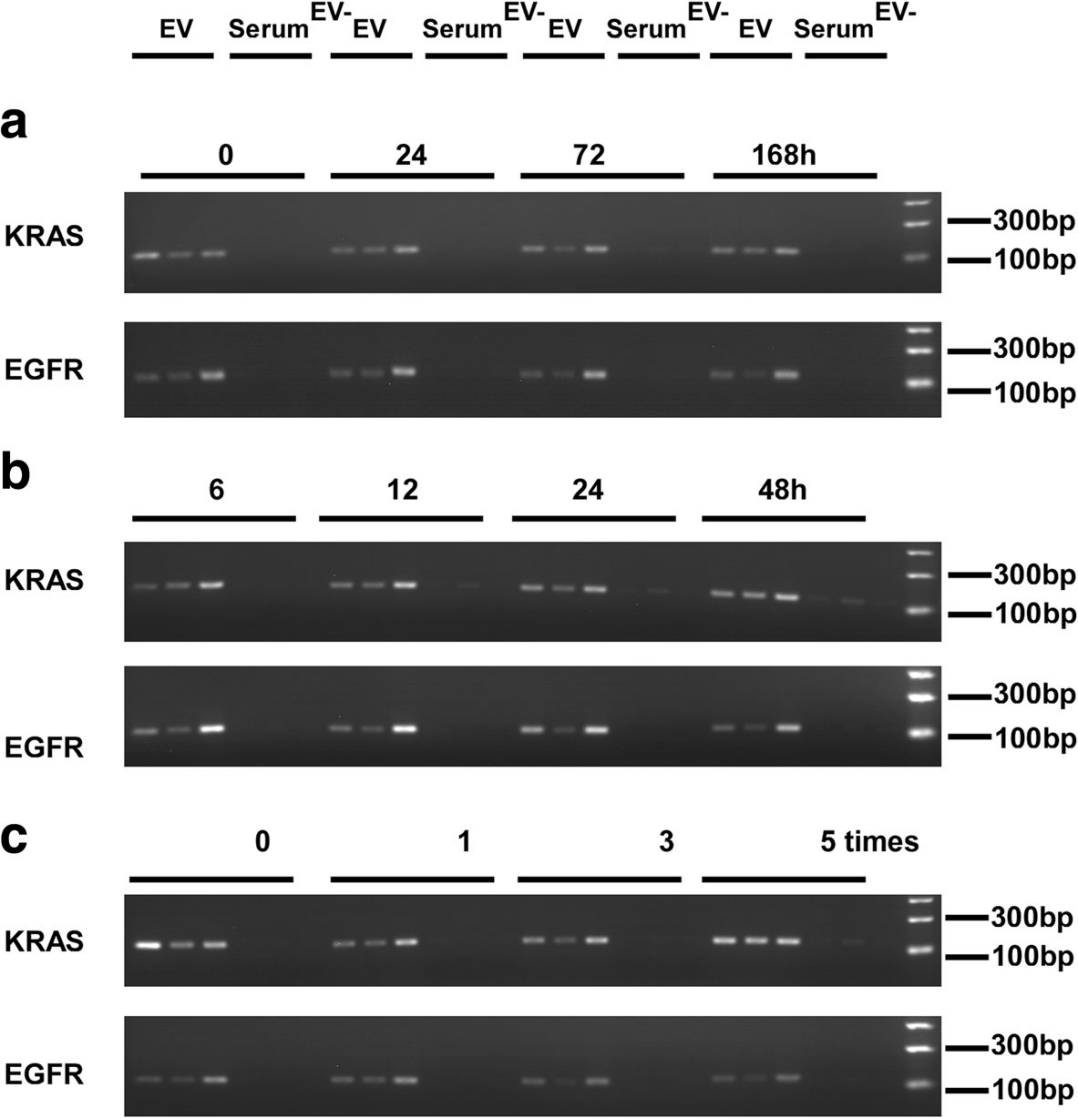
Amplification of *KRAS* and *EGFR* in EVs and EVs-depleted serum under different conditions. **a** *KRAS* and *EGFR* were amplified in EVs and EVs-depleted serum from the serum of three patients stored at 4 °C for 0, 24, 72 and 168 h. **b** *KRAS* and *EGFR* were amplified in EVs and EVs-depleted serum from the serum of three patients stored at room temperature for 0, 6, 12, 24 and 48 h. **c** *KRAS* and *EGFR* were amplified in EVs and EVs-depleted serum from the serum of three patients after repeated freeze-thaw 0, 1, 3 and five times. Serum^EV-^ represents EVs-depleted serum

The observed changes that occurred in PCR (polymerase chain reaction) products were consistent with the EVs DNA concentration (Fig. 5). EVs DNA stayed stable for 1 week at 4 °C (Fig. 5a), 1 day at room temperature (Fig. 5b) and repeated freeze-thaw three cycles (Fig. 5c), even though there were slight differences among patients and primers. Also, the PCR products of EVs-depleted serum gradually increased, accompanied by vesicle rupture under a variety of extreme conditions. In summary, these results show that EVs DNA is extremely stable, especially at 4 °C.

### EVs provides an attractive means for gene detection

**Fig. 6:**
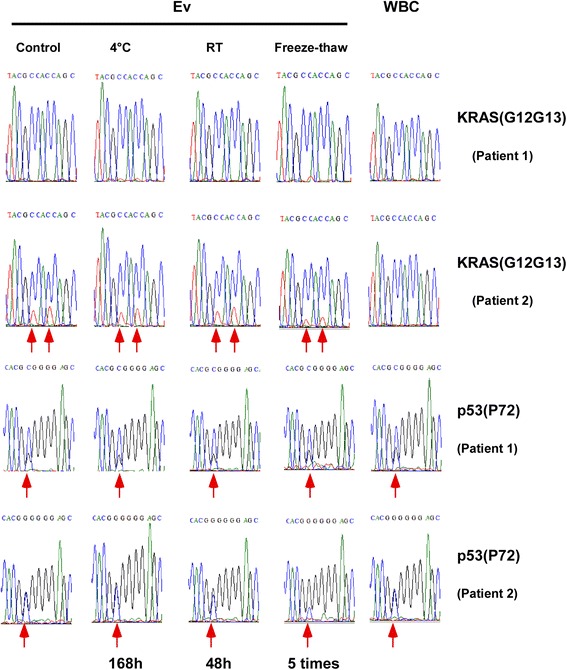
Sanger sequencing of EVs DNA extracted from serum under different conditions. Sanger sequencing revealed a *p53* mutation on codon 72 in EVs DNA from patient 1, a *p53* mutation on codon 72 and two *KRAS* mutations on codon 12 and 13 in EVs DNA from patient 2; white blood cell (WBC) DNA from the same patient was used as a control

We collected serum samples from two CRC patients. By histological examination, we determined that one CRC patient had a mutated form of the *KRAS* gene and the other patient was wild-type. The extraction conditions of the EVs were as follows: 0 h after the blood was centrifuged, 4 °C for 168 h, room temperature for 48 h and five freeze-thaw cycles. EVs DNA was isolated from serum that was stored under the above conditions, and the *KRAS* and *p53* PCR products were sequenced (Table 1). Simultaneously, we extracted the leukocytes from these two CRC patients to use for control. Our results showed that PCR amplification from EVs DNA was successful regardless of storage conditions (Fig. 6). The sequencing results showed no significant difference among the different storage conditions compared with the freshly prepared samples (Fig. 6). Moreover, after the 5-time freeze-thaw cycle, there was a significant decrease in the sequencing peak values in the samples containing the mutant *KRAS* gene locus and the wild-type *KRAS* locus (Fig. 6). Therefore, we conclude that the repeated freeze-thaw condition has the largest impact on the stability of EVs. However, no matter what condition was analyzed, the *KRAS* mutation was detected in the EVs DNA, although the sequencing peak value was different (Fig. 6). Furthermore, the genetic testing results between exosomes and tumor cells showed a high degree of consistency. Patient tumor DNA that showed a mutation in exon 2 of the *KRAS* gene by histological examination showed the same mutation by sequencing in the patient’s EVs DNA. Moreover, no mutations were detected in the somatic cells of patients. In summary, our results suggest that exosomes can be used as an ideal material for gene detection.

## Discussion

Cancer is one of the leading causes of morbidity and mortality in the world, and the treatment of it has evolved during the past two decades. Early diagnosing and effective treatment can significantly reduce cancer mortality, which relies on accurate diagnosis, precise tumor staging, and gene mutation status [37]. As molecular targeted therapy develops, standardized treatment of tumors should depend on the results of gene detection [38]. Genetic testing of tumor tissues is the current gold standard for cancer diagnosis and therapy. Primary lesions and metastatic foci in early tumors are hard to find. Furthermore, as cancer advances, surgery and biopsy is rather difficult, and they are both invasive techniques, which could bring more suffering to the patient. Importantly, results of genetic tests to help guide treatment are not necessarily accurate because tumor tissue is heterogeneous [39]. Therefore, with our current in-depth study of EVs (especially exosomes), we ushered in the dawn to solve this problem.

Exosomes from different cell types have different functions, due to the composition of their functional molecules, which varies from their cell of origin [40–42]. Exosomes contain multiple proteins, DNA, mRNA, microRNA, long noncoding RNA and genetic material from viruses/prions [43]. These substances are biochemically and functionally distinct when transferred to a recipient cell where they regulate protein expression and signaling pathways [44]. Tumor-derived exosomes play a significant role in facilitating metastases by strengthening angiogenesis [45]. Evs are emerging as key players in intercellular communication between cancer cells and their microenvironment through horizontal transfer of information via their cargo [25, 46]. Meanwhile, the more noteworthy fact is, serum exosomes from cancer patients contain genomic DNA that spans all chromosomes [29], and that exosomes from cancer patients have a higher concentration than normal cells [47, 48]. It is well-known that the stability of exosomes depends largely on their bilayer lipid membrane structure to protect biological cargo against degradation and denaturation in the extracellular environment [49]. The focus of our work is for the stability of EVs DNA and possibility of being a novel approach for genetic testing.

In the present study, we tested the stability of serum EVs by analyzing their molecular markers, DNA concentration, PCR amplification, and sequencing from exoDNA. The membrane bilayer structure is the basis for the stability of the exosome; therefore, the exoDNA can be protected from degradation. Similar to mRNA [50], the exoDNA content remains substantially at a constant level after experiencing different treatment conditions. EVs experience a slight rupture with the length of time and increasing the number or freeze-thaw cycles. We observed an apparent change in DNA concentration from 24 to 48 h at room temperature. However, the change was not significant on the initial concentration. Nevertheless, the largest degradation of EVs appeared during the freeze-thaw cycles, and subsequent sequencing results also reflect this. Similarly, by DNA electrophoresis we showed that with the deterioration of conditions, such as 4 °C for 168 h, room temperature for 48 h and repeated freezing and thawing five times, the serum without intact EVs began to show increasing amounts of DNA products, which implies the rupture of EVs. Also, primer differences exist between different patients under various conditions. Importantly, exosomes are recognized as promising diagnostic and predictive biomarkers in cancer. Therefore, clinicians might use them to guide individual treatments [51]. Our research confirmed that EVs or exosomes exhibit extremely stable characteristics and can protect themselves from material damage by the external environment. EVs show a considerable degree of consistency with tumor cells on the detection of genetic mutation. Therefore EVs may be a suitable choice for genetic testing and/or guiding the individual treatment of a patient.

## Conclusions

In conclusion, Serum EVs, and EVs DNA can remain stable under different storage environments, which is the premise that EVs could be serve as a novel means for tumor genetic detection and potential biomarkers for cancer diagnostics and prognostics. Furthermore, maybe we can monitor changes in EVs gene during patients treatment to better guide individualized therapy.

## Abbreviations

cfDNA: cell free DNA
CRC: colorectal cancer
ctDNA: circulating tumor DNA
dsDNA: double-stranded DNA
EVs: Extracellular vesicles
exoDNA: exosomal DNA
mCRC: metastatic colorectal cancer
mRNA: messenger RNA
mtDNA: mitochondrial DNA
MVB: Multivesicular bodies
ncRNA: noncoding RNA
PBS: Phosphate buffer saline
PCR: Polymerase chain reaction
pdna: plasmid DNA
PM: Plasma membrane
PVDF: Polyvinylidene fluoride
ssDNA: single-stranded DNA
